# Engineering Pyranose 2-Oxidase for Modified Oxygen Reactivity

**DOI:** 10.1371/journal.pone.0109242

**Published:** 2014-10-08

**Authors:** Dagmar Brugger, Iris Krondorfer, Christopher Shelswell, Benjamin Huber-Dittes, Dietmar Haltrich, Clemens K. Peterbauer

**Affiliations:** Food Biotechnology Laboratory, Department of Food Sciences and Technology, BOKU - University of Natural Resources and Life Sciences, Vienna, Austria; Instituto de Tecnologica Química e Biológica, UNL, Portugal

## Abstract

Pyranose 2-oxidase (POx), a member of the GMC family of flavoproteins, catalyzes the regioselective oxidation of aldopyranoses at position C2 to the corresponding 2-ketoaldoses. During the first half-reaction, FAD is reduced to FADH_2_ and reoxidized in the second half-reaction by reducing molecular oxygen to H_2_O_2_. Alternative electron acceptors including quinones, radicals or chelated metal ions show significant and in some cases even higher activity. While oxygen as cheap and abundantly available electron acceptor is favored for many processes, reduced oxygen reactivity is desirable for some applications such as in biosensors/biofuel cells because of reduced oxidative damages to the biocatalyst from concomitant H_2_O_2_ production as well as reduced electron “leakage” to oxygen. The reactivity of flavoproteins with oxygen is of considerable scientific interest, and the determinants of oxygen activation and reactivity are the subject of numerous studies. We applied site-saturation mutagenesis on a set of eleven amino acids around the active site based on the crystal structure of the enzyme. Using microtiter plate screening assays with peroxidase/2,2′-azino-bis(3-ethylbenzthiazoline-6-sulphonic acid) and 2,6-dichlorophenolindophenol, variants of POx with decreased oxidase activity and maintained dehydrogenase activity were identified. Variants T166R, Q448H, L545C, L547R and N593C were characterized with respect to their apparent steady-state constants with oxygen and the alternative electron acceptors DCPIP, 1,4-benzoquinone and ferricenium ion, and the effect of the mutations was rationalized based on structural properties.

## Introduction

Pyranose 2-oxidase (POx; pyranose:oxygen 2-oxidoreductase; synonym, glucose 2-oxidase; EC1.1.3.10) from *Trametes multicolor* (*Tm*POx; synonym, *Trametes ochracea* [1]) is a member of the glucose-methanol-choline (GMC) family of oxidoreductases [2]–[4]. POx is an intracellular enzyme but is encountered in the culture medium upon prolonged cultivation, probably due to hyphal autolysis [3]. The homotetrameric *Tm*POx has a molecular mass of 270 kDa [1], and each subunit binds flavin adenine dinucleotide (FAD) covalently as a co-factor *via* its 8α-methyl group to N^ε2^ (i.e. N3) of His^167^ [5], [6]. POx contributes to the ligninolytic system as a producer of H_2_O_2_ for lignin-degrading peroxidases [7], [8] and is involved in the quinone redox cycling machinery [1]. POx catalyzes the regioselective oxidation of aldopyranoses, preferrentially D-glucose [1], at position C2 and the corresponding 2-ketoaldoses are formed [9]. During this first half reaction FAD is reduced to FADH_2_ (eq1). The ensuing half reaction reoxidizes the cofactor by reducing molecular oxygen to H_2_O_2_ (eq2) [10]. Possible alternative electron acceptors include quinones (eq3), radicals or chelated metal ions [1]. This reaction catalyzed by POx is typically found in flavoprotein oxidoreductases [11], [12] and is of the Ping Pong Bi Bi type [13].
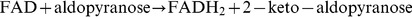
(1)

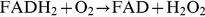
(2)

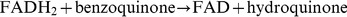
(3)


In terms of catalytic efficiency some of the alternative electron acceptors are in fact better substrates for the enzyme than oxygen [1], [14], but until now the chemical and structural reasons for reactivity with either electron acceptor are unidentified [15]. Flavoproteins can be classified based on their reactivity with molecular oxygen [11], [16], [17]: *Electron transferases* produce significant amounts of O_2_^−^ [18] and form the neutral flavin semiquinone over the whole range of their pH stability [19], whereas *dehydrogenases* generate variable, and often very low, amounts of O_2_^−^ when reacting with O_2_ [18]. *Oxidases* show a rapid second order reaction of the reduced enzyme with oxygen and thermodynamically stabilize an anionic flavin semiquinone [11], [16]. The common characteristic of the enzyme group of *monooxygenases* is the easy formation and stabilization of flavin C(4a)-flavin hydroperoxide when reacting with O_2_ [20], which can also be observed for POx [13], [21] and pyranose dehydrogenase [22]. The reaction of oxygen with reduced flavin was described as follows [17], [23]: one e^−^ is transferred from singlet reduced flavin Fl_red_ H^−^ (↑↓) to triplet O_2_ (↑↑) and forms a caged radical pair [FLH^•^↑↑O_2_^−^]. This radical pair can follow several reaction paths [17], [24]: (i) it can dissociate to the oxygen radical O_2_^−^; (ii) a second e^−^ can be added and H_2_O_2_ as well as oxidized flavin Fl_ox_ are produced; (iii) spin inversion [FLH^•^↑↓O_2_^−^] leads to C(4a)-flavin hydroperoxide formation FlHOO^−^ + H^+^ ↔ FlHOOH, which is unstable in aqueous solution and decays to H_2_O_2_ and oxidized flavin Fl_ox_ or inserts an oxygen atom into a substrate. The transfer of the first electron from the 2-e^−^ reduced flavin to oxygen is thought to be rate limiting [17], [24]–[26]. The resulting flavin semiquinone is unstable but could be detected by for glycolate oxidase [27]. The environment around the C4a-N5 locus, which is directly involved in the reaction with oxygen, is discussed to be the crucial area affecting oxidase activity [28]. A positive charge around the flavin reaction site, which can be provided by amino acid residues [15], [26], [29]–[32] or bound substrate and/or product [33]–[36], can influence this. Channels leading from the surface to the active site may affect oxidase reactivity [37], [38]. Altering the oxygen reactivity is of considerable interest as shown in recent reviews [15], [28] and works of Krondorfer et al. [39], Sygmund et al. [40], Leferink et al. [41], and others [42]–[44].

In this work we aimed at identifying amino acid residues that influence oxygen reactivity of PO using a semi-rational protein engineering approach. Based on the crystal structure of the enzyme [6], a set of eleven amino acids around the active site was chosen as target positions for site-saturation mutagenesis [45]. Using microtiter plate screening assays with peroxidase/2,2′-azino-bis(3-ethylbenzthiazoline-6-sulphonic acid) (ABTS) and 2,6-dichlorophenolindophenol (DCPIP), variants of *Tm*POx with decreased oxidase activity and maintained dehydrogenase activity were identified. Five selected variants were shown to possess decreased oxygen reactivity and conserved activity with alternative electron acceptors like DCPIP, 1,4-benzoquinone (1,4-BQ) and ferricenium ion (Fc^+^) by determination of the apparent steady-state constants.

## Results

### Site saturation mutagenesis and screening of mutant libraries

**Figure 1:**
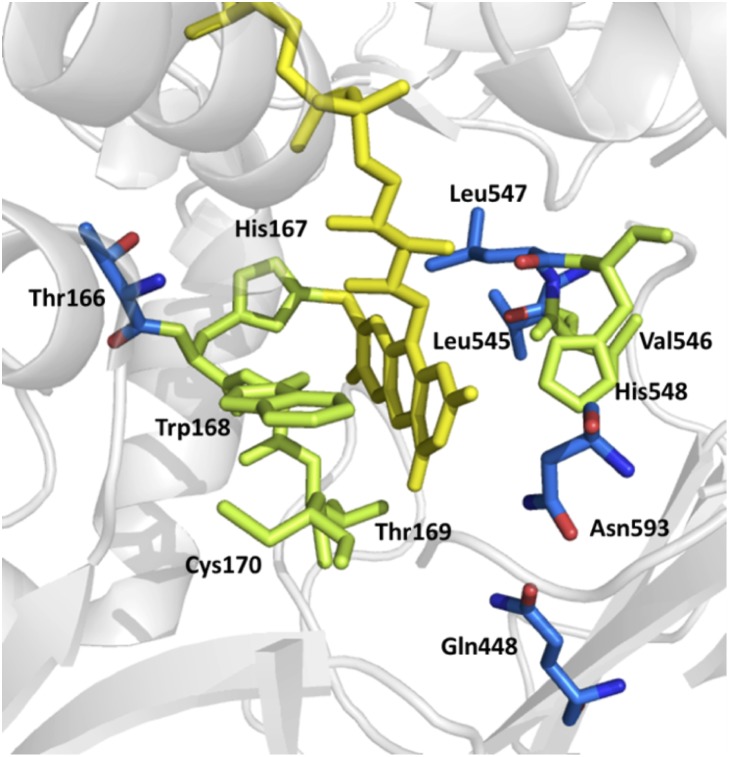
Active site residues of *T. multicolor* POx selected for saturation mutagenesis. FAD is shown in yellow, residues in blue show variants with decreased oxygen activity. The image was generated with PyMOL using coordinates from PDB 1tt0, for clarity not all residues are shown.

Target positions for site-saturated mutagenesis were eleven residues surrounding the flavin cofactor (Thr^166^, His^167^, Trp^168^, Thr^169^, Cys^170^, Gln^448^, Leu^545^, Val^546^, Leu^547^, His^548^ and Asn^593^; Fig. 1). Generation of mutants, expression in *E. coli* BL21 star DE3 and screening of POx variants in 96-well plates were carried out as described in the Material and Methods section. To cover more than 95% of all possible variants [46], 172 colonies were screened for position Trp^168^, Cys^170^, Gln^448^ and Leu^547^, and 344 colonies were investigated for all other positions (Thr^166^, His^167^, Thr^169^, Leu^545^, Val^546^, His^548^ and Asn^593^). Screening of POx variants was performed using the standard assay (peroxidase/ABTS assay) employing 100 mM D-glucose as electron donor, oxygen (air) as electron acceptor, and following the formation of the ABTS radical from the produced H_2_O_2_ by horseradish peroxidase [47]. An assay with 100 mM D-glucose as electron donor and DCPIP as alternative electron acceptor was performed in parallel [48]. Colonies showing higher absorption changes than the negative control in either the peroxidase/ABTS assay or the DCPIP assay were considered active. Thr^169^ is the most conserved of the investigated amino acid positions, with 64% inactive colonies. At positions His^167^, Cys^170^, Leu^547^, His^548^ and Asn^593^ the active/inactive distribution is well balanced with 47%, 42%, 54%, 56% and 47% active variants, respectively. Changes at positions Thr^166^, Trp^168^, Gln^448^, Leu^545^ and Val^546^ produce a high number of active variants (70%, 74%, 84%, 75% and 88%). In all positions a large majority of active variants (more than 74% in all positions, more than 84% in all but two) showed activity with both electron acceptors (Table S1 in File S1). We did not identify a position where mutations abolish either oxidase or dehydrogenase activity entirely, and only three positions (Cys^170^, Leu^545^ and Val^546^) showed either one activity being more frequent than the other by a factor of more than five. A number of variants were cultivated in small shaken flasks, the enzyme purified and again subjected to the two standard assays (Table S2 in File S1). A large part of these variants were identified as having significantly reduced activity with oxygen as well as DCPIP, ranging from 0.1% to 8% of wild type activity. No variant was identified that had strongly reduced dehydrogenase activity and unaltered oxidase activity. A large number of variants that initially appeared to have residual DCPIP activity but no oxygen activity in the plate screening turned out to be “false positives” and actually showed strongly reduced activity with both electron acceptors when tested with purified enzymes rather than lysates in the plate screening. Five variants showing less than 5% oxidase activity (peroxidase/ABTS assay) and at least 50% of dehydrogenase activity (DCPIP assay) compared to the wild type were selected for further study.

### Protein expression, purification and kinetic characterization

**Figure 2:**
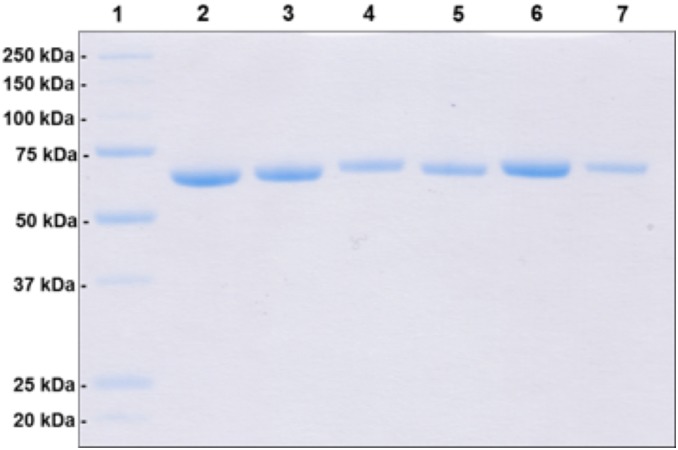
SDS-PAGE of wild-type POx and variants. *Lane 1*, molecular mass standard Precision Plus Protein Unstained (BioRad); *lane 2*, wild type POx; *lane 3*, T166R; *lane 4*, Q448H; *lane 5*, L545C; *lane 6*, L547R; *lane 7*, N593C.

Cultures of transformed *E. coli* expressing the POx variants T166R, Q448H, L545C, L547R and N593C were cultivated in shaken flasks. POx variants were purified from the crude cell extract using IMAC, concentrated and washed (50 mM phosphate buffer pH 6.5) by ultrafiltration. The apparent homogeneity was confirmed by SDS-PAGE (Fig. 2), and the purified variants T166R, Q448H, L545C, L547R and N593C were characterized with respect to their oxidase and dehydrogenase activity. Oxidase activity was determined by following the consumption of oxygen using an Oxygen Microsensor. The two-electron two-proton acceptor DCPIP, the two-electron one-proton acceptor 1,4-BQ and the one-electron acceptor Fc^+^ were used to evaluate the dehydrogenase activity in order to avoid a possible bias.

#### Kinetic parameters for oxygen catalyzed reaction

**Figure 3:**
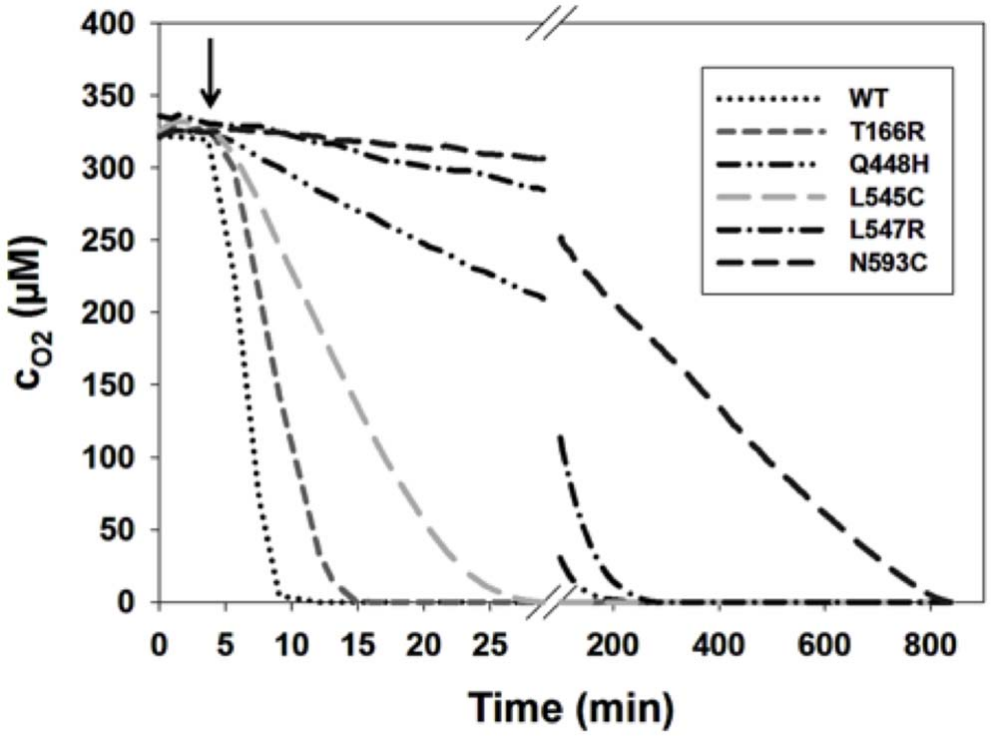
Oxygen consumption of wild-type and various POx variants. Measurements were carried out at 25°C in 50 mM phosphate buffer (pH 6.5) with 100 mM D-glucose as electron acceptor. The arrow indicates addition of POx (0.02 mg). The reduction of oxygen concentration was followed with an Oxygen microsensor. The assay contained 2000 U catalase to remove hydrogen peroxide. **⋅⋅⋅**, wt; – –, T166R; – **⋅⋅** –, Q448H; – –, L545C; – ⋅ –, L547R; – –, N593C.

The apparent steady-state kinetic parameters for oxidase activity are summarized in Table 1. L547R and N593C show considerable changes with both the largest increases in *K*_m_ (3 and 7.5-fold) and dramatic decreases in *k*_cat_ (by 97% and 99.5%, respectively), resulting in a catalytic efficiency of approximately one and 0.1% of wild-type POx. The other three selected variants show 39% (T166R), 2% (Q448H) and 19% (L545C) of wild-type *k*_cat_/*K*_m_ values with oxygen as electron acceptor, all resulting from decreased turnover numbers at marginally changed or unchanged *K*_m_ values. Fig. 3 depicts the consumption of oxygen of wild-type POx and the variants T166R, Q448H, L545C, L547R and N593C, employed at identical quantities. The consumption of dissolved oxygen took 7 min for wild-type POx, 11 min for T166R, 24 min for L545C, 243 min for Q448H, 277 min for L547R and 834 min for N593C.

**Table 1:** Apparent steady-state kinetic constants of wild-type and mutant POx from with D-glucose (100 mM) as electron donor and O_2_ (varied from 5–400 µM) as electron acceptor.

	Oxidase activity			
Variant	*K*_m_ (mM)	*k*_cat_ (s^−1^)	*k*_cat_/*K*_m_ (mM^−1 ^s^−1^)	Relative *k*_cat_/*K*_m_ (%)
WT	0.13±0.02	42±2	308±41	100
T166R	0.092±0.015	11±1	120±22	39
Q448H	0.082±0.018	0.38±0.03	4.6±1.0	1.5
L545C	0.046±0.006	2.7±0.1	58±8	19
L547R	0.40±0.12	1.1±0.2	2.8±0.9	0.99
N593C	0.98±0.55	0.18±0.07	0.18±0.07	0.058

#### Kinetic constants for the electron acceptors DCPIP, 1,4-BQ and Fc^+^

The data summarized in Tables 2, 3 and 4 represent the dehydrogenase activity with DCPIP, 1,4-BQ and Fc^+^ of the five selected variants compared to wild-type POx. More information about the reaction mechanism of the alternative electron acceptors is provided in File S1 and [49]. T166R and L547R show increased apparent turnover numbers for the DCPIP-mediated dehydrogenase activity whereas the *K*_m_ values remain in the wild-type range (Table 2). The amino acid exchanges in Q448H and N593C affect both *K*_m_ (eight- and tenfold increases, respectively) and *k*_cat_ (decreases by 88% and 94%, respectively). All variants except N593C display enhanced or constant catalytic efficiencies with DCPIP as electron acceptor in comparison to the wild-type.

**Table 2:** Apparent steady-state kinetic constants of wild-type and mutant POx with D-glucose as electron donor (100 mM) and DCPIP (varied from 0.015–1.2 mM) as electron acceptor.

	DCPIP-mediated dehydrogenase activity			
Variant	*K*_m_ (mM)	*k*_cat_ (s^−1^)	*k*_cat_/*K*_m_ (mM^−1 ^s^−1^)	Relative *k*_cat_/*K*_m_ (%)
WT	1.9±0.1	70±4	38±3	100
T166R	2.1±0.5	265±47	127±38	334
Q448H	0.24±0.03	8.1±0.4	34±5	90
L545C	0.72±0.07	50±2	70±7	184
L547R	1.7±0.4	134±20	79±22	208
N593C	0.18±0.01	3.9±0.1	22±1	58

**Table 3:** Apparent steady-state kinetic constants of wild-type and mutant POx with D-glucose as electron donor (100 mM) and 1,4-BQ (varied from 0.01–0.5 mM) as electron acceptor.

	1,4-BQ-mediated dehydrogenase activity			
Variant	*K*_m_ (mM)	*k*_cat_ (s^−1^)	*k*_cat_/*K*_m_ (mM^−1 ^s^−1^)	Relative *k*_cat_/*K*_m_ (%)
WT	0.24±0.03*	152±6*	632±83*	100
T166R	0.14±0.02	166±11	1213±191	192
Q448H	0.057±0.007	12±1	203±30	32
L545C	0.042±0.006	110±4	2608±384	413
L547R	0.018±0.003	26±1	1465±251	232
N593C	0.14±0.02	4.6±0.2	32±5	5.1

*data from reference [47].

**Table 4:** Apparent steady-state kinetic constants of wild-type and mutant POx with D-glucose as electron donor (100 mM) and Fc^+^ (varied from 0.005–0.5 mM) as electron acceptor.

	Fc^+^-mediated dehydrogenase activity			
Variant	*K*_m_ (mM)	*k*_cat_ (s^−1^)	*k*_cat_/*K*_m_ (mM^−1 ^s^−1^)	Relative *k*_cat_/*K*_m_ (%)
WT*	0.25±0.10*	151±35*	592±274*	100
T166R	1.6±0.4	442±79	281±86	48
Q448H	0.56±0.21	27±7	48±22	8.1
L545C	0.12±0.01	142±4	1193±105	202
L547R	0.23±0.01	134±3	584±29	98
N593C	0.81±0.20	22±4	27±8	4.6

*data from reference [47].

The affinity to 1,4-BQ as expressed by the Michaelis constant increased for all five mutants, most notably for L545C and L547R (Table 3), whereas the apparent *k*_cat_ value is decreased for all variants except T166R. The former two show 4.1-fold and 2.3-fold increases in catalytic efficiency, despite the observed decrease in turnover number. Again N593C showed the most significant decrease in catalytic efficiency.

Variants Q448H and N593C again showed strongly reduced catalytic efficiencies with the one-electron acceptor Fc^+^, due to both increased *K*_m_ values and reduced turnover numbers. L545C and L547R show improved and unaltered catalytic efficiencies, respectively, due to an improved *K*_m_ value for the former and almost unaffected turnover numbers (Table 4).

**Figure 4:**
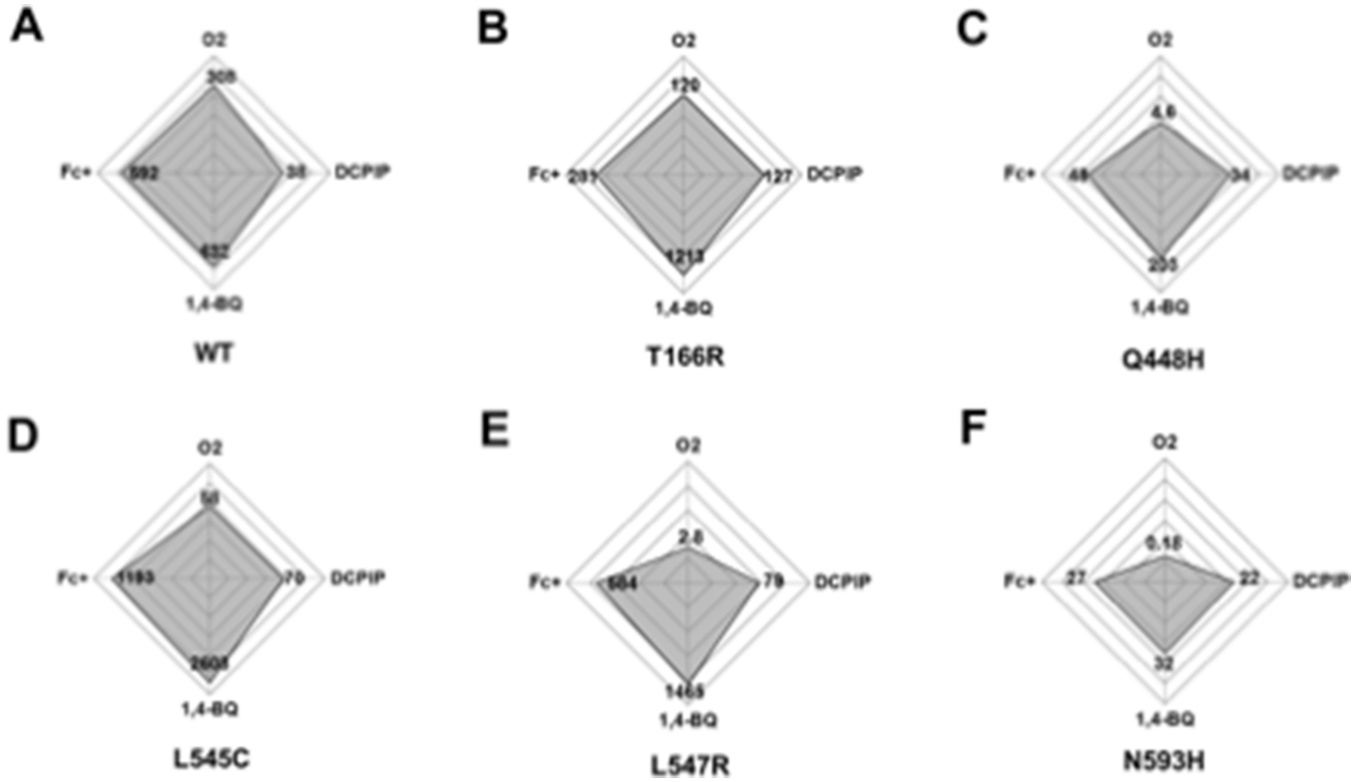
Radar plots illustrating the catalytic efficiency of oxidase and dehydrogenase activity. (oxygen consumption) (DCPIP, 1,4-BQ and Fc^+^ as electron acceptors) of (A) wild-type POx, as well as variants (B) T166R, (C) Q448H, (D) L545C, (E) L547R and (F) N593C. The axes denote *k*_cat_/*K*_m_ values in logarithmic scale from 0.01 to 10000 mM^−1 ^s^−1^. Plots were prepared using Excel 2010 (Microsoft).

The *k*_cat_/*K*_m_ values of each mutational variant for the different electron acceptors are given in radar plots for better visualization of the experimental results (Fig. 4). Radar graphs allow the simultaneous evaluation of the overall activity of a variant (distance to the center) and the substrate usage (shape of the plot) [45], [50]. Each axis illustrates the catalytic efficiency constant determined by using oxygen, as well as DCPIP, 1,4-BQ and Fc^+^ as electron acceptor. For comparison wild-type POx was included (Fig. 4A) and clear changes in the pattern between the wild-type and the variants T166R, Q448H, L545C, L547R and N593C are evident. The amino acid substitutions in the variants cause lower activity toward oxygen compared to the wild-type, which turns the shapes of the charts from rhombic (wild-type) to deltoid (variants), even though different activities are mapped. From the plots it is obvious that the variants show increased preferences for the electron acceptors DCPIP, 1,4-BQ and Fc^+^ in relation to oxygen compared to the wild-type. 1,4-BQ is the favored electron acceptor for both variants and wild-type, followed by Fc^+^.

**Figure 5:**
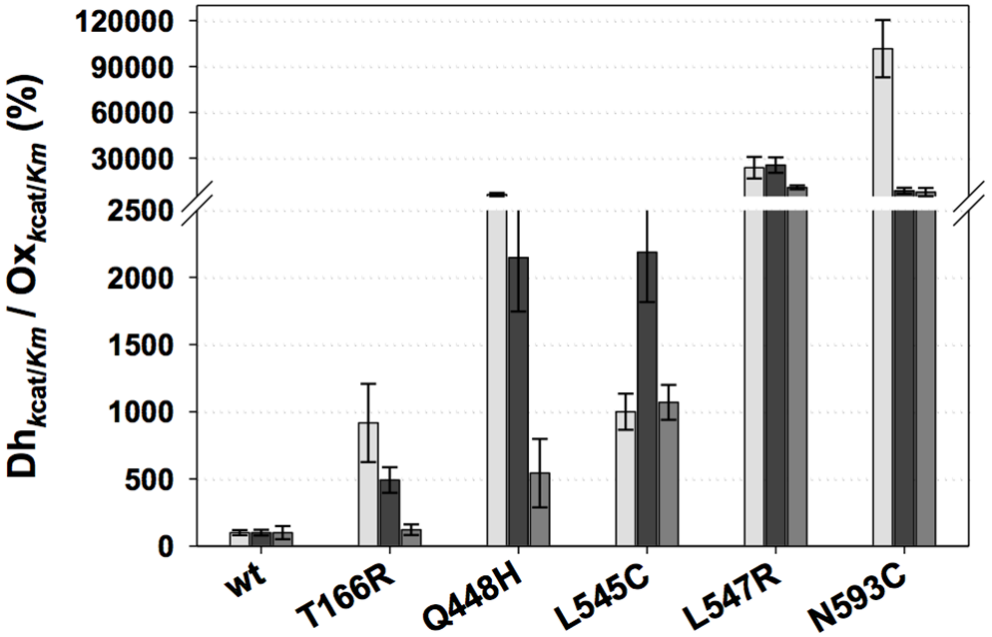
Dehydrogenase/Oxidase ratios of *k*_cat_/*K*_m_ of POx variants compared to the wild-type. Dehydrogenase activity was determined using DCPIP (light grey bars), 1,4-BQ (dark grey bars) and Fc^+^ (mid grey bars) as electron acceptors, oxidase activity was determined by following oxygen consumption. Wild-type activities were set at 100%. D-glucose was used as electron donor at 100 mM. Results are the mean of at least duplicate independent experiments and error bars correspond to standard deviations. The diagram was prepared using SigmaPlot 11.

This preference for the alternative electron acceptors over oxygen is further expressed by the substrate specificity values, *i.e*., the ratio of the specificity constants *k*_cat_/K_m_ for the two substrates [51]. All variants show increased ratios of Dehydrogenase (Dh*_k_*_cat/*K*m_) to Oxidase (Ox*_k_*_cat/*K*m_) catalytic efficiency compared to wild-type POx, regardless of the electron acceptor used (Fig. 5). The largest and most balanced improvement is shown by L547R, with an increase of substrate specificity values by a factor of 240, 250 and 110 for DCPIP, 1,4-BQ and Fc^+^, respectively. The single most dramatic increase is seen in the amino acid exchange at position 593 from Asn to Cys, resulting in a 1000-fold increased dehydrogenase/oxidase ratio with DCPIP as electron acceptor. This results from the drastically decreased oxidase activity (0.1%) at 59% residual activity with DCPIP. The Dh*_k_*_cat/*K*m_/Ox*_k_*_cat/*K*m_ ratio is increased 80-fold for 1,4-BQ and Fc^+^. T166R, Q448H and L545C show increased dehydrogenase/oxidase ratios in the range of 1.2 to 9-fold (T166R), 5 to 61-fold (Q448H) and 10 to 22-fold (L545C).

## Discussion

Pyranose 2-oxidase is a well investigated oxidoreductase for industrial applications in the area of food technology and biosensors/biofuel cells [14], [52]–[55]. Reduced oxygen reactivity is a desirable property for some applications because of reduced oxidative damages to the biocatalyst from concomitant H_2_O_2_ production, and also for applications in biosensors/biofuel cells because of reduced “leakage” of electrons to oxygen and a more quantitative transfer to the redox mediator or electrode surface. The reactivity of flavoproteins with oxygen is of considerable scientific interest, and the determinants of oxygen activation and reactivity are the subject of numerous studies [39]–[44]. The consensus is that there is no single parameter (like spatial accessibility, charge, hydrophobicity, vicinity of particular amino acid residues etc.) determining whether a flavoprotein is (primarily) an oxidase or a dehydrogenase, but rather a set of parameters influencing the environment around the cofactor and the substrate in ways that are still incompletely understood. In the absence of clear structural determinants for (predominant) oxidase or dehydrogenase activity, we applied site saturation mutagenesis to eleven amino acid residues in the vicinity to the active site of POx and screened for reduced oxidase and unaltered dehydrogenase activities.

POx contains an internal void of roughly 15,000 Å and all four active sites open up only to this cavity [3]. A substrate molecule entering the active site has to pass through one of four channels leading from the surface to the void and then has to overcome the flexible active site loop [6], [49]. Thr^166^ is part of the amino acid sequence motif ^165^STHW^168^ related to flavinylation [6]. This residue is rather distant from the flavin (11.7 Å), and both the decrease in oxidase activity (to 39%) as well as the increase in dehydrogenase activity caused by the exchange of the small, neutral and polar Thr for the large, basic and polar Arg were moderate except for DCPIP (threefold increase). Other variants at this position (Phe, Tyr) show strongly reduced activity with both oxygen and DCPIP to less than 2% of wild type and no “shift” towards dehydrogenase activity (Table S1 in File S1). The same observation was made with variants at other positions of this motif as well as the neighboring positions Thr^169^ and Cys^170^, where T169H retains substantial activity with both electron acceptors (19 and 14%, respectively), but again no “dehydrogenase shift” is observed – initial indications in the plate screening, particularly for position Cys^170^, could not be confirmed when purified proteins were analyzed.

Q448H also shows significantly reduced oxidase activity (2% of the wild-type) and almost unaltered activity with DCPIP as electron acceptor, resulting in a shifted oxidase/dehydrogenase ratio. This is not uniformly valid, as dehydrogenase activity also dropped with the other alternative electron acceptors 1,4-BQ and Fc^+^. The considerable effect with the charged electron acceptor Fc^+^ could result from repulsion through an additional positive charge in the active site. Other variants at this position such as Q448K or Q448R again generally affect both types of activity (Table S2 in File S1).

Leu^545^ and Leu^547^ on the *si*-side of the flavin are part of a leucine triad, located at the entry from the void to the active site [6]. The change of the hydrophobic amino acid Leu to the small, polar Cys at position 545 and to the basic, polar Arg at position 547 leads to a significantly decreased oxidase activity (19% and 1%, respectively) without losing dehydrogenase activity almost irrespective of the alternative electron acceptor. In addition, the introduction of another positively charged side chain as in Q448H (distance to N5-C(4a) 7.4 Å) reduced *k*_cat_/*K*_ox_ by a factor of 67, while dehydrogenase activity was maintained or only slightly reduced with DCPIP or 1,4-BQ. Oxygen-reacting flavoenzymes often carry positively charged groups near N5 of the isoalloxazine to enhance the reaction with oxygen [31], and to date histidine, lysine or a charged product formed at the active site have been shown to act as such an electrostatic catalyst [33]. At first glance our results seem to contradict other reports, since the introduction of a positive charge greatly reduced reactivity with oxygen. An explanation could be that residues 545 and 547 are not in immediate vicinity of the N5-C(4a) locus (distance 12 and 8 Å), and influence oxygen reactivity indirectly through sterical/structural changes of neighboring residues, e.g. His^548^, which has been proposed as both the catalytic base [56] and involved in the protonation process to form the C(4a)-hydroperoxy-flavin intermediate in the oxidative half reaction [57]. Nonpolar residues close to the flavin C(4a) [33] or in the access channel to the active site [43] have been identified together with the above mentioned positive charges in several flavoprotein oxidases, and the exchange against the polar amino acids Cys and Arg may contribute to reduced oxygen accessibility to the active site and decreased activity.

Together with His^548^, Asn^593^ is involved in the reductive half reaction by forming a productive enzyme-substrate complex [6] and transferring electrons from the sugar substrate to the flavin. This stabilizing pair (either His-His or His-Asn) is known from other members of the GMC family like PDH, CDH, ChOx and GOx [22], [58]–[60]. The importance of this amino acid, which is also closest to the flavin C(4a) (4.5 Å) is illustrated by the significant reductions in catalytic efficiency with all electron acceptors except for DCPIP (58% residual activity). The strong “shift” towards dehydrogenase activity with DCPIP is rather caused by the dramatic reduction in the activity with oxygen and a favorable Michaelis constant, and it has to be noted that this variant is significantly hampered in catalytic performance to a point where an application in biocatalysis probably is not a prospect anymore. It is interesting to note that essentially for all investigated positions, only one amino acid exchange could be identified to have affected oxidase activity selectively, whereas all other mutations affected both activities. From all variants that were purified, only two, T166F and H167P, showed dehydrogenase activity that was significantly stronger reduced than oxidase activity (by a factor of approximately six, Table SI.2). This suggests that dehydrogenase activity is somewhat more “robust” than oxidase activity. Since oxygen-utilizing enzymes appeared later in evolution than dehydrogenases, which already existed in an atmosphere devoid of molecular oxygen, this is not surprising.

We recently pursued a similar objective in the catalytically related pyranose dehydrogenase, an enzyme that reacts extremely slowly with oxygen, namely to substantially increase oxidase activity, either with unaltered or at the expense of dehydrogenase activity [39]. In this work, which followed an essentially identical approach of substituting amino acid residues in proximity of the flavin C(4a) by site-saturation mutagenesis, we identified only one residue that influenced oxidase activity to a measurable degree: His103, carrying the covalently attached flavin cofactor, with the observed increase in oxygen reactivity most likely due to changes in the redox potential of the cofactor [39].

The results of this and the presented work confirm that oxygen activation/reactivity is a property influenced by a number of features of the enzymés active site architecture. Structural accessibility, polarity, hydrophobicity and charge combine intricately to generate an environment favorable for oxygen activation and electron transfer from the flavin cofactor to oxygen. This delicate balance can be upset by small alterations such as the amino acid exchanges described here, resulting in significantly reduced oxidase activity. It seems, however, much more difficult to generate such a favorable environment by exchanges of single or a few amino acid residues. Presumably a more radical and thorough re-engineering of the entire substrate-binding and cofactor-binding architecture is required to introduce oxygen reactivity. The amino acid positions identified in our study will be of interest for further studies using e.g. rapid kinetic experiments to elucidate the effect on the individual half reactions in detail. In addition, it will be interesting to study the effect of combinations of some of the above described mutations, in order to further decrease oxygen reactivity while maintaining dehydrogenase activity with alternative electron acceptors. Gene shuffling between mutated parent genes or simply introducing an additional mutation by site-directed mutagenesis into a variant gene offers reasonably quick access to such double- or triple-mutated variants. A combination of, e.g., either L545C or L547R with Q448H (which maintains high dehydrogenase activity only with DCPIP, but not 1,4-BQ and Fc^+^) may not result in a variant with universally high dehydrogenase activity, and the same may be true for combinations with mutations at the catalytically important amino acid N593 (which all display significantly reduced kinetic constants, with DCPIP-mediated dehydrogenase activity as the only exception). The combination of L545C and L547R, both introducing polar amino acids close to the *si*-side of the flavin, however, appears promising, if two mutations this close together do not interfere with foldability and stability. A combination of T166R, also increasing polarity near the flavin cofactor and moderately affecting both oxidase and dehydrogenase activity, with either of these mutations or both, also appears attractive in order to obtain additive or even synergistic effects of these mutations. In addition, the here described variants can be used as template for subjecting selected positions in the neighborhood of the replaced amino acids to saturation mutagenesis. Such approaches will be the subject of further studies.

## Materials and Methods

### Chemicals

All chemicals were of the highest purity available and purchased from Sigma Aldrich (St. Louis, MO) or Merck (Darmstadt, Germany) unless otherwise stated. Molecular mass marker and Bio-Safe Coomassie stain for sodium dodecyl sulphate-polyacrylamide gel electrophoresis (SDS-PAGE) were purchased from BioRad (Hercules, CA).

### Bacterial strains, plasmids and media

C-terminal His_6_-tagged POx gene from *T. multicolor* was expressed in electro-competent *E. coli* BL21 Star DE3 cells (Thermo Fisher Scientific Biosciences, Waltham, MA) using the pET21d^+^/POx (pHL2) vector as described in [61]. Cells were grown on LB_amp_ plates (10 g L^−1^ peptone from casein, 5 g L^−1^ yeast extract, 10 g L^−1^ NaCl, 14 g L^−1^ agar supplemented with 100 µg mL^−1^ ampicillin) and plasmid pHL2 was used as template for all mutagenic PCRs. Production of recombinant POx was carried out using TB_amp_ medium (12 g L^−1^ peptone from casein, 24 g L^−1^ yeast extract, 4 mL L^−1^ glycerol, 100 µg mL^−1^ ampicillin, KH_2_PO_4_-buffer 1 M, pH 7).

### Generation of mutants

Site-saturation mutagenesis was used for the generation of POx libraries encoding mutants that contain all proteinogenic amino acids at the target positions. The plasmid pHL2 containing the wild-type gene was used as template. The applied overlap extension method was described by Ho et al. [62]. PCRs were carried out using Phusion high-fidelity DNA polymerase from New England BioLabs (Ipswich, MA), deoxynucleoside triphosphates (dNTP) from Thermo Fisher Scientific and oligonucleotide primers from VBC Biotech (Vienna, Austria). Flanking primers T7_fwd/T7_rev and internal mutagenic oligonucleotide primers containing the variable position NNS (N…A, C, G or T and S…G or C) were used for the amplification of two DNA fragments with overlapping end-regions. Details to the used primers are listed in Table S3 in File S1. Subsequently, DNA fragments were fused by an additional PCR (primers T7_fwd/T7_rev) to create the mutated full-length gene. PCR products were separated by agarose gel electrophoresis and purified using the Wizard SV Gel and PCR-Clean-Up System (Promega, Madison, WI). Mutated POx genes (inserts) and pHL2 vector (backbone) were digested with restriction endonucleases *Not*I/*Nco*I and ligated using T4 DNA ligase (Thermo Fisher Scientific Biosciences). Five µL of plasmid DNA were transformed into electro-competent *E. coli* BL21 Star DE3 cells. The presence of the mutations was verified by DNA sequencing (VBC Biotech) using the T7_fwd and T7_rev primer.

### Screening assay in 96-well plates

Mutant libraries were transferred from LB_amp_ plates into standard 96-well plates. When using oligonucleotide primers of the NNS type 95 colonies have to be screened to cover all possible variants of the target position with 95% probability [46]. Six wells per plate were inoculated with *E. coli* BL21 Star DE3 cells carrying the wild-type plasmid pHL2 (positive control) and four wells per plate with *E. coli* BL21 Star DE3 cells harbouring the pET21d^+^ vector without POx gene (negative control). Cultivation, expression and sample preparation of the libraries were carried out according to Spadiut et al. [47] without freezing step after cell lysis. The supernatant was used for the activity assay with peroxidase/ABTS and DCPIP as described in [47] and [48] with modifications: 20 µL of supernatant were mixed with 80 µL ABTS reaction mix (0.035 mg mL^−1^ horseradish peroxidase, 0.7 mg mL^−1^ in 50 mM phosphate buffer pH 6.5 and 100 mM D-glucose), or with 80 µL DCPIP reagent containing 0.054 mg mL^−1^ DCPIP in 50 mM phosphate buffer pH 6.5 and 100 mM D-glucose. The formation of ABTS^+•^ was followed at 420 nm and DCPIP reduction was detected at 620 nm with a Sunrise™ microplate reader (Tecan, Männedorf, CH).

### Protein expression and purification

Wild-type POx and selected variants were cultivated and purified as described previously [14]. *E. coli* BL21 Star DE3 cells, harboring pET21d^+^/POx wild-type or mutant plasmid, were grown in 1 L TB_amp_ medium (four flasks with 250 mL each) at 37°C and 160 rpm until reaching an OD_600_ of 0.5. After induction of expression with 0.5% w/v lactose incubation continued for further 20 h at 25°C and 160 rpm. After centrifugation (6000 rpm, 30 min, 4°C; Avanti J-26XP, Beckman-Coulter, Brea, CA) the harvested cell pellet was resuspended in 50 mM phosphate buffer (pH 6.5) containing 1 g L^−1^ PMSF (phenyl methyl sulfonyl fluoride), and cells were broken up using a precooled homogenizer (APV Systems, Gatwick, UK). The crude extract was obtained by ultra-centrifugation (25000 rpm, 30 min, 4°C) and loaded onto 20 mL Profinity IMAC Ni-Charged Resin (Bio-Rad). After washing the column with 20 column volumes of 50 mM phosphate buffer pH 6.5 with 500 mM NaCl, proteins were eluted with a linear gradient (50 mM phosphate buffer, 500 mM NaCl, 1 M imidazole, pH 6.5). Active fractions were concentrated and washed by ultrafiltration using an Amicon Ultra Centrifugal Filter Device with a 30 and 100-kDa cut-off membrane (Millipore; Billerica, MA).

### Protein analysis

Protein concentration of purified enzyme was determined by Bradford’s method using the BioRad Protein Assay Kit with bovine serum albumin as standard. SDS-PAGE was performed with Precision Plus Protein Unstained as mass standard and stained with Bio-Safe Coomassie stain (both from Biorad).

### Enzyme activity assay

To determine the activity of POx wild-type and mutants the standard peroxidase/ABTS assay was performed [63], [64]. Ten µL of diluted enzyme were added to 980 µL of assay buffer containing horseradish peroxidase (142 U) and ABTS (14.7 mg) in phosphate buffer (50 mM, pH 6.5). The reaction was started by adding 20 mM D-glucose. Absorbance change at 420 nm was recorded at 30°C for 180 sec (ε_420_ = 43.2 mM^−1 ^cm^−1^). One unit of POx activity was defined as the amount of enzyme needed for the oxidation of 2 µmol of ABTS per min under assay conditions. Additionally POx activity was measured following the time dependent reduction of 150 µM DCPIP (two-electron acceptor) in 50 mM phosphate buffer (pH 6.5) containing 20 mM D-glucose at 520 nm and 30°C (ε_520 nm_ = 6.8 mM^−1 ^cm^−1^) [65]. The reaction was started by adding 10 µL of diluted POx sample. A Beckman-Coulter DU 800 photometer was used to determine the standard activity with peroxidase/ABTS, DCPIP and the protein concentration.

### Kinetic constants for reaction with oxygen and measurement of oxygen consumption

Apparent kinetic constants for oxygen were determined as described previously [40], [66]: Initial rates of oxygen consumption were measured at 25°C in a 1.5 mL screw neck vial (VWR International, Radnor, PA) sealed with a septum. The vial was filled with 50 mM phosphate buffer (pH 6.5) containing 100 mM D-glucose and mixed with a magnetic stirrer. The phosphate buffer was treated with oxygen or nitrogen to achieve dissolved oxygen concentrations between 5 and 400 µmol L^−1^. The Oxygen Microsensor (PreSens GmbH, Regensburg, Germany) was inserted through the septum and the reaction was started by adding a suitable amount of enzyme using a Hamilton syringe. Oxygen consumption was recorded and initial velocity values were calculated from linear plots of apparent O_2_ concentration versus time.

To compare oxygen consumption of the variants with the wild-type, 0.02 mg of each protein was applied. Additionally 2000 U bovine liver catalase (Fluka/Sigma Aldrich) were added to the reaction mix to prevent POx enzyme damage caused by hydrogen peroxide production during the reaction.

### Determination of kinetic constants for the reaction with DCPIP, 1,4-BQ and Fc^+^

Kinetic constants were determined for the two-electron two-proton acceptor DCPIP, the two-electron one-proton acceptor 1,4-BQ, and the one-electron acceptor Fc^+^ ion). All measurements were carried out in 50 mM phosphate buffer (pH 6.5) and reactions were followed for 180 s using a Lambda 35 UV/VIS spectrophotometer or a U-3000 spectrophotometer (Hitachi, Tokyo, Japan). 1,4-BQ and Fc^+^ assays were recorded at 30°C whereas DCPIP reduction was followed at room temperature (22°C). Depending on the wavelength and the electron acceptor concentration disposable plastic cuvettes (10 mm path length; Greiner Bio One, Kremsmünster, Austria) or quartz cuvettes (3 or 10 mm path length; Hellma Analytics, Müllheim, Germany) were used. For DCPIP, 1,4-BQ and Fc^+^ assays 100 mM D-glucose was used as electron donor, and the concentration of electron acceptors was varied between 0.015–1.2 mM for DCPIP, 0.01–0.5 mM for 1,4-BQ and 0.005–0.5 mM for Fc^+^. Initial rates of DCPIP, 1,4-BQ and Fc^+^ reduction were recorded at 520, 290 and 300 nm, and the corresponding molar extinction coefficients are ε_520 nm_ = 6.8 mM^−1^ cm^−1^, ε_290 nm_ = 2.24 mM^−1 ^cm^−1^ and ε_300 nm_ = 4.3 mM^−1 ^cm^−1^. Apparent kinetic constants were calculated by non-linear least-square regression, fitting the data to the Henri-Michaelis-Menten equation, using SigmaPlot (Systat Software Inc., San Jose, CA). Turnover numbers were calculated assuming a molecular mass of 68 kDa for the POx subunits. All measurements were performed at least in duplicates.

## Supporting Information

File S1 Contains the following files: **Table S1.** Activity distribution in the 96-well plate screening. Clones that produced a higher absorbance in the microtiter plate wells as described in Material and Methods than the negative control were considered active and grouped according to their activity with either of the two electron acceptors ABTS and DCPIP, or both. **Table S2.** Oxidase and dehydrogenase activity of IMAC-purified variants relative to the wild type. **Table S3.** Nucleotide sequences of primers used in saturation mutagenesis. **Scheme S1.** Reaction mechanism of the alternative two-electron two-proton acceptor DCPIP. Prepared with Chemograph plus 6.4.
(DOCX)
