# Supplementary material for: Engineering Pyranose 2-Oxidase for Modified Oxygen Reactivity
Source: PLoS One. 2014 Oct 8;9(10):e109242. doi: 10.1371/journal.pone.0109242 (PMC4190269; doi:10.1371/journal.pone.0109242)
Supplement: File S1 — Contains the following files: Table S1. Activity distribution in the 96-well plate screening. Clones that produced a higher absorbance in the microtiter plate wells as described in Material and Methods than the negative control were considered active and grouped according to their activity with either of the two electron acceptors ABTS and DCPIP, or both. Table S2. Oxidase and dehydrogenase activity of IMAC-purified variants relative to the wild type. Table S3. Nucleotide sequences of primers used in saturation mutagenesis. Scheme S1. Reaction mechanism of the alternative two-electron two-proton acceptor DCPIP. Prepared with Chemograph plus 6.4. (DOCX) [file pone.0109242.s001.docx]

| **Position** | **ABTS + DCPIP (%)** | **ABTS (%)** | **DCPIP (%)** |
| --- | --- | --- | --- |
| T166 | 95 | 1 | 4 |
| H167 | 86 | 10 | 4 |
| W168 | 94 | 1 | 5 |
| T169 | 80 | 12 | 8 |
| C170 | 74 | 3 | 23 |
| Q448 | 86 | 10 | 4 |
| L545 | 84 | 15 | 1 |
| V546 | 92 | 1 | 7 |
| L547 | 84 | 11 | 5 |
| H548 | 88 | 5 | 7 |
| N593 | 84 | 8 | 8 |

**Table SI.2.** Oxidase and dehydrogenase activity of IMAC-purified variants relative to the wild type.

| **Variant** | **Oxidase activity (%)** | **Dehydrogenase activity (%)** |
| --- | --- | --- |
| **WT** | **100.0** | **100.0** |
| T166F | 1.5 | 0.2 |
| T166Y | 0.1 | 0.8 |
| **T166R** | **15.2** | **33.3** |
| H167P | 1.9 | 0.3 |
| H167I | 0.8 | 0.3 |
| W168L | 0.0 | 0.2 |
| W168E | 0.5 | 0.3 |
| W168G | 0.0 | 0.0 |
| W168Q | 0.1 | 0.2 |
| T169H | 19.1 | 13.7 |
| T169P | 1.0 | 0.9 |
| C170F | 0.1 | 0.2 |
| C170A | 0.0 | 0.3 |
| C170Y | 0.0 | 0.3 |
| Q448K | 0.1 | 0.2 |
| Q448R | 0.1 | 0.8 |
| **Q448H** | **3.3** | **10.2** |
| L545Q | 0.0 | 0.3 |
| **L545C** | **53.8** | **189.6** |
| V546G | 0.0 | 6.5 |
| V546L | 1.7 | 8.2 |
| V546F | 0.0 | 0.3 |
| V546W | 0.1 | 0.4 |
| **L547R** | **7.7** | **259.1** |
| H548L | 0.2 | 0.3 |
| H548C | 0.9 | 0.4 |
| H548N | 0.5 | 0.0 |
| H548R | 0.1 | 1.3 |
| N593E | 0.3 | 2.5 |
| **N593C** | **0.5** | **19.8** |

**Table SI.3.** Nucleotide sequences of primers used in saturation mutagenesis

| Primer | Sequence 5’ – 3’ |
| --- | --- |
| T166wobble_fwd | catgtctnnscactggacatgcg |
| T166wobble_rev | atgtccagtgsnnagacatgcctccgag |
| H167wobble_fwd | atgtctacgnnstggacatgcgccac |
| H167wobble_rev | tgtccasnncgtagacatgcctccg |
| W168wobble_fwd | tgtctacgcacnnsacatgcgccacacc |
| W168wobble_rev | gtgtggcgcatgtsnngtgcgtagac |
| T169wobble_fwd | gtctacgcactggnnstgcgccacaccc |
| T169wobble_rev | gtgtggcgcasnnccagtgcgtag |
| C170wobble_fwd | tctacgcactggacannsgccacaccc |
| C170wobble_rev | gggtgtggcsnntgtccagtgcg |
| Q448wobble_fwd | ccgtggcacactnnsatccaccgcgat |
| Q448wobble_rev | atcgcggtggatsnnagtgtgccacgg |
| L545wobble_fwd | gagcctggtnnsgtccttcacctt |
| L545wobble_rev | aaggtgaaggacsnnaccaggctcc |
| V546wobble_fwd | gagcctggtcttnnscttcaccttgg |
| V546wobble_rev | ccaaggtgaagsnnaagaccaggctcc |
| L547wobble_fwd | ctggtcttgtcnnscaccttggtggtacg |
| L547wobble_rev | taccaccaaggtgsnngacaagaccaggc |
| H548wobble_fwd | agcctggtcttgtccttnnscttggtggtacgc |
| H548wobble_rev | gcgtaccaccaagsnnaaggacaagaccaggct |
| N593wobble_fwd | cgtacggcgcgnnsccgacgctcaccg |
| N593w_rev | cggtgagcgtcggsnncgcgccgtacgc |
| T7prom | taatacgactcactatag |
| T7term | gctagttattgctcagcgg |

**Scheme SI1:** Reaction mechanism of the alternative two-electron two-proton acceptor DCPIP. Scheme prepared with Chemograph plus 6.4.
